# An Exploratory Pilot Study on Choking Episodes in Archery

**DOI:** 10.3389/fpsyg.2021.585477

**Published:** 2021-02-12

**Authors:** Pierluigi Diotaiuti, Stefano Corrado, Stefania Mancone, Lavinia Falese, Fábio Hech Dominski, Alexandro Andrade

**Affiliations:** ^1^Department of Human, Social and Health Sciences, University of Cassino, Cassino, Italy; ^2^Santa Catarina State University, Florianopolis, Brazil

**Keywords:** archery, sport anxiety, choking under pressure, coping styles, decentering, coach support

## Abstract

The aim of our study is to test the fit of an explanatory model of the frequency of the phenomenon of choking under pressure in archers, focusing on both the individual components (anxiety, coping styles, and decentralization) and environmental components (perception of coach assistance). 115 competitive athletes including 72 males (62.6%) and 43 females (37.4%) participated in the study, with average age of 39 years (±15.47). Participants reported personal data and completed measures of self-consciousness, anxiety, coping styles, and decentering. The ruminative component of concern was found to be the factor directly influencing the frequency of choking episodes in performance. Two significant mediations of personal coping style were also identified on the effects of anxiety on brooding thinking and on the athlete's ability to decentralize. The latter personal self-regulative component has been shown to be able to strongly limit the frequency of choking under pressure. Finally, among the environmental components, a further facilitating influence for the athlete resulted in the perception of being supported by the coach. The results therefore suggest that the athlete in choking should not face alone the hard upcoming period, but should preferably be supported with an articulated program focused on the cognitive remodeling of disturbing thoughts, on the strengthening of the capacity of decentralization, on the enhancement of the relationship of support and trust with the coach.

## Introduction

Archery is a sport that consists in firing an arrow toward a target as precisely as possible, in which fine motor precision skills are required for success. In order to have high accuracy, there is a need of a good technique, tactic, specific physical conditions and psychology conditions, such as: motivation, confidence, anxiety control, self-control, tenacity to overcome any pressure and concentration (Humaid, 2014).

Recent studies have conducted research on psychological factors in sports, showing the influence of psychological aspects on athletes' performance (Brandt et al., 2014, 2017; Andrade et al., 2016). In that context, the target panic is a block in archery performance (Prior and Coates, 2019) characterized by both physical symptoms like dystonia and psychological symptoms like choking (Clarke et al., 2015), and manifests as a stiffening of the archer's body, which is no longer able to execute the correct movement, for e.g., the arm of the arch is blocked, there is a difficulty to perform a correct anchorage, problems to exit the clicker correctly, a difficulty in releasing the arrow, an inability to put the sight pin on the gold or an inability to release the arrow at the appropriate time (the release is anticipated or delayed). The archer that experiences a choking phenomenon is usually worried about being able to maintain a steady posture to shot, doubts his abilities, tries to exasperate the control of his shot and experiences symptoms of somatic anxiety (increased heart rate, muscular tension) and cognitive anxiety (worry, apprehension) (Weinberg and Gould, 2003).

Masters (1992) assumed that choking occurred due to increased pressure, while for Drinan et al. (2000) choking is the combined result of lack of attention and anxiety and it involves both with a debilitating effect due to anxiety and an improper placement of attention (Masters, 1992; Drinan et al., 2000). Wang (2002) definition implies that choking occurs only in athletes who have reached a level of “performing routine processes”, while for Baumeister and Showers even novices could experience choking (Baumeister and Showers, 1986; Wang, 2002).

Physiological aspects of the human movement and psycho-neuromuscular impediment known as “yips” may cause performance problems and are also related to the phenomenon of choking under pressure (Smith et al., 2000). There is evidence that choking-affected athletes showed higher levels of anxiety sensitivity originating from physical, cognitive and social sources (Clarke et al., 2019).

In the last 20 years researchers have made serious attempts to understand choking, proposing different perspectives, analyzing personality characteristics and models to determine mechanisms for choking, but none of them is universally accepted. In many scientific papers choking is defined as the occurrence of suboptimal performance in pressure situations (Baumeister, 1984; Hill et al., 2010; Gröpel and Mesagno, 2017). In sport, choking under pressure is the failure for athletes in meeting self-imposed performance expectations in critical situations due to a combination of factors that increases anxiety and includes features such as competition, the presence of audience, reward or punishment contingency, and ego relevance (Baumeister and Showers, 1986; Mesagno and Beckmann, 2017).

Among the potential moderators of choking in sport, coping styles have also to be mentioned. The scientific literature suggests that an approach coping style and/or an escapist self-regulatory coping style (Wang et al., 2004; Jordet, 2009; Hill et al., 2010) increase the possibility of choking.

The phenomenon of yips and choking has been studied mainly in golf (Clarke et al., 2015), but also in sports like basketball (Fryer et al., 2018; Gómez et al., 2018; Morgulev and Galily, 2018), tennis (Iwatsuki et al., 2018), handball (Debanne et al., 2018), soccer (Jordet et al., 2012) and other team sports (Wergin et al., 2018) but, as far as we know, there are few studies for archery.

When choking under pressure affects an athlete, the target panic can occur, especially in sport with throws, shots and target aiming. For this reason, it is especially interesting to study sports where the athlete has to focus on a target or on a throw.

Indeed, there is no doubt that the mental component and personality traits play a fundamental role in this problem, but it is not clear the mechanism behind this process. Moreover, further research is needed to investigate if anxiety and attention act concurrently, or if one precedes or follows the other.

This study aims to investigate whether individual components (anxiety, coping styles, and decentralization) and environmental components (perception of coach assistance) influence the frequency of the phenomenon of choking under pressure in archers.

## Methods

### Study Design

This is a cross-sectional study analyzing the phenomenon of choking under pressure in archers.

### Participants

Participants were 115 competitive athletes including 72 males (62.6%) and 43 females (37.4%). The average age was 39 years (SD = 15.47) with an interval ranging from 10 to 74 years. The sampling took place in a randomized form within the members of the *Italian Archery Federation* belonging to Lazio Region (total 2,325 athletes). The subjects freely participated in the study, signing the consent to the confidential treatment of the data in aggregate form only for the scientific purpose.

### Procedure

A questionnaire structured into a general information section and a psychometric section was administered in June and July 2019. The tool envisaged the possibility of filling in online by using a specific link (https://my.questbase.com/take.aspx?pin=8183-7258-7800) and collecting data on the *Questbase* platform. Participants were recruited directly during official Italian archery competitions. The questionnaire was anonymous, and the subjects were informed that the use was only for research purposes. All approached athletes gave written informed consent in accordance with the Declaration of Helsinki.

### Instruments

The *general section* of questionnaire included the following data: (1) personal data (gender, age, year of completion of the basic course); (2) class to which they belong (masters, seniors, juniors, students); (3) category (first, second, third, fourth); (4) arch preference (compound, Olympic, nude, long bow); (5) preference for competitive locations (indoor, outdoor); (6) training preference (alone, groups); (7) weekly training sessions; (8) duration of training (in hours); (9) perceived support by the coach in training and competition; (10) maximum level of competition (last year); (11) frequency choking episodes in the last 6 months.

The *psychometric section* included the following tools:

1. *Self-Consciousness Questionnaire* (SCQ) (Fenigstein et al., 1975; revised version 1985), Italian version (Comunian, 1994). It includes 21 multiple-choice items (Likert 1–4 from *no identification* to *full recognition*). Three main subscales: Private Self-Awareness (orientation to reflection and concentration on the Self); Public self-awareness (orientation to reflection and concentration on others); Social anxiety (difficulty and tension in the relationship and interaction with others; fear of external judgment). Scales reliability with Cronbach's alpha resulted 0.79 for Social anxiety, 0.70 for Public Self-awareness; 0.73. for Private Self-awareness.
2. *The Sport Anxiety Scale* (SAS) (Smith et al., 2006). Includes 21 multiple choice items (Likert 1–4 from *at all* to *very*) three subscales: Pre-occupation (personal doubts, performance fears, fear of failure) with Cronbach's alpha 0.90; Somatic anxiety (nervousness, tension, palpitations) with Cronbach's alpha 0.92; Concentration Disorder (distractions, inattentiveness, drop in concentration) with Cronbach's alpha 0.73.
3. *Coping Style Inventory for Athletes* (CSIA) (Anshel and Kaissidis, 1997), consists of 16 multiple-choice items (Likert 1–5 from *never* to *always*); two sub-scales: *Approach Style* (task reinvestment strategies) with a scale reliability measurement for Cronbach's alpha: 0.68 and *Avoidance Style* (emotional control strategies, challenge rationalization) with scale reliability measurement for Cronbach's alpha: 0.70.
4. *Decentering Scale for Sport* (DSS) (Zhang et al., 2016) consists of 12 Likert 1–5 items (from never to always). The scale evaluates the subject's ability to detach himself cognitively from the involvement of strong and unpleasant emotional pressures; emotional regulation. The factorial analysis of the main components confirmed the monofactorial structure of the instrument, with Cronbach's alpha 0.78.

## Statistical Analysis

The data were processed using the statistical software SPSS version 22 and Amos IBM version 22. The tests performed were: descriptive statistics, Pearson and Spearman's bivariate correlations, *T*-tests, univariate Anova, hierarchical regression, path analysis structural equations, mediation analysis. The latter was performed through the PROCESS macro version 2.3 (www.processmacro.org; Hayes, 2017). To test the adequacy of the SEM model were considered the following eight indices: (1) the chi-square; (2) the relationship between the value of the chi-square and the degrees of freedom; (3) GFI (Goodness of Fit Index); (4) AGFI (Adjusted Goodness of Fit Index); 5) RMSEA (Root-Mean-Square Error of Approximation); 6) RMSR (Root Mean Square Residual); 7) CFI (Comparative Fit Index); 8) NFI (Normed Fit Index); 9) RFI (Relative Fit Index); 10) PNFI (Parsimony Adjustment to NFI); 11) PCFI (Parsimony Adjustment to CFI); 12) PCLOSE (testing the null hypothesis that the population RMSEA is no > 0.05).

## Results

### Descriptive Analysis

On average the archers had 11 years of experience since the end of the basic course (SD = 9.30) with an interval ranging from 1 to 42 years. The average of weekly training sessions was 3.22 (SD = 1.39); for an average duration of 2.32 h per session (SD = 0.78). Table 1 shows the sample characteristics (class and category of competition, type of bow, preference for competition locations, training preference, and level of competition).

**Table 1:** Sample characteristics.

**Class of competition**	***n* (%)**
Masters	32 (27.8%)
Seniors	66 (57.4%)
Juniors	8 (7%)
Students	9 (7.8%)
**Category of competition**	
First (highest)	39 (33.9%)
Second	18 (15.7%)
Third	34 (29.6%)
Fourth (lowest)	24 (20.9%)
**Type of bow**	
Olympic arc	68 (59.1%)
Compound	26 (22.6%)
Naked bow	18 (15.7%)
Longbow	3 (2.6%)
**Preference for competitive locations**	
Outdoor race	72 (62.6%)
Indoor race	43 (37.4%)
**Training preference**	
Groups	65 (56.5%)
Alone	50 (43.5%)
**Level of competition**	
Interregional level	33 (28.7%)
Regional level	35 (30.4%)
Nationally	34 (29.6%)
Internationally	13 (11.3%)

Considering the frequency of choking under pressure in the last 6 months, the whole sample reported an average number of 3.08 episodes, with a minimum of one and maximum of seven episodes. The average duration of choking, expressed in training sessions was 6.02 (SD = 9.75), while the maximum duration recorded (even outside the 6 months considered) was 15.57 episodes (SD = 33.73). Out of 34 (29.6%) participants stated that the period of the questionnaire submission coincided with a choking interval. Following Table 2 reports the bivariate correlations among main variables of the study.

**Table 2:** Bivariate correlations.

	**CHO**	**DEC**	**SOCA**	**SOMA**	**CODI**	**APP**	**AVO**	**PRE**	**PRSA**	**PUSA**	**CCS**	**TCS**
CHO	1											
DEC	−0.354^**^	1										
SOCA	0.126	−0.352^**^	1									
SOMA	0.241^**^	−0.439^**^	0.425^**^	1								
CODI	0.266^**^	0.402^**^	0.307^**^	0.536^**^	1							
APP	−0.327^**^	0.376^**^	−0.116	−0.034	−0.173	1						
AVO	−0.140	0.339^**^	−0.201^*^	−0.222^*^	−0.162	0.266^**^	1					
PRE	0.303^**^	−0.497	0.511^**^	0.596^**^	0.584^**^	−0.582^**^	−0.366^**^	1				
PRSA	0.161	0.042	0.017	0.075	0.077	−0.143	0.037	0.110	1			
PUSA	0.234^*^	−0.416^**^	0.722^**^	0.374^**^	0.302^**^	−0.422^**^	−0.288^**^	0.570^**^	0.098	1		
CCS	−0.151^*^	−0.102	0.021	0.102	−0.036	0.048	−0.069	0.159	0.175	0.015	1	
TCS	−0.095	−0.149	−0.087	−0.022	0.016	0.017	−0.028	0.137	0.085	−0.032	0.703^**^	1

*CHO, Choking; DEC, Decentering; SOCA, Social Anxiety; SOMA, Somatic Anxiety; CODI, Concentration Disturbance; APP, Approach Style; AVO, Avoidance Style; PRE, Pre-occupation. PRSA, Private Self-Awareness; PUSA, Public Self-Awareness; CCS, Competition Coach Support; TCS, Training Coach Support; N, 115. For Choking Spearman's coefficient was used*.

** Correlation is significant at the 0.01 level (2-tailed);

* *Correlation is significant at the 0.05 level (2-tailed)*.

### Differences in Relation to Gender

Female archers reported significantly higher levels of Pre-occupation: T (113) = −2.262; *p* < 0.05 Sig. = 0.03; M_male_ = 2.93 (DS = 1.34); M_female_ = 3.51 (DS = 1.40); and lower values for Decentralization: *T*_(113)_ = 2.007; *p* < 0.05 Sig. = 0.04; M_male_ = 3.40 (DS = 1.92); M_female_ = 2.75 (DS = 1.47).

## Effects of Anxieties and Coping Styles on the Archer's Pre-Occupation

A hierarchical multiple regression was run to determine if the addition of components of Anxiety and Coping Styles improved the prediction of the Athlete's Pre-occupation. The preliminary verifications of the regression assumptions excluded the presence of multivariate outliers. Mardia's multivariate kurtosis index (84.49) was in fact below the critical value [p (p+2) = 90]; therefore the relationship between the variables can be considered substantially linear. Low co-linearity was indicated by the low VIF values (Variance Inflation Factor) <2 and high tolerance values > 0.60. For verification of the assumptions on the residuals, the average between the standardized and raw residuals was equal to 0; the Durbin–Watson test had a value of 1.58 and was therefore indicative of the absence of autocorrelation.

As regards the Pre-occupation, influential predictors have been identified in *Somatic Anxiety* (β = 0.216; Δ*R*^2^ = 0.355), *Approach Style* (β = −0.322; Δ*R*^2^ = 0.164), *Concentration Disorder* (β = 0.295; Δ*R*^2^ = 0.069), *Social Anxiety* (β = 0.189; Δ*R*^2^ = 0.031), and *Avoidance Style* (β = −0.147; Δ*R*^2^ = 0.020). The full model to predict *Athlete's Pre-occupation* was statistically significant, *R*^2^ = 0.638, *F*_(5, 114)_ = 38.483, *p* < 0.0005; adjusted *R*^2^ = 0.622.

## Coping Styles as Mediators of Anxieties on the Athlete's Pre-Occupation

**Figure 1:**
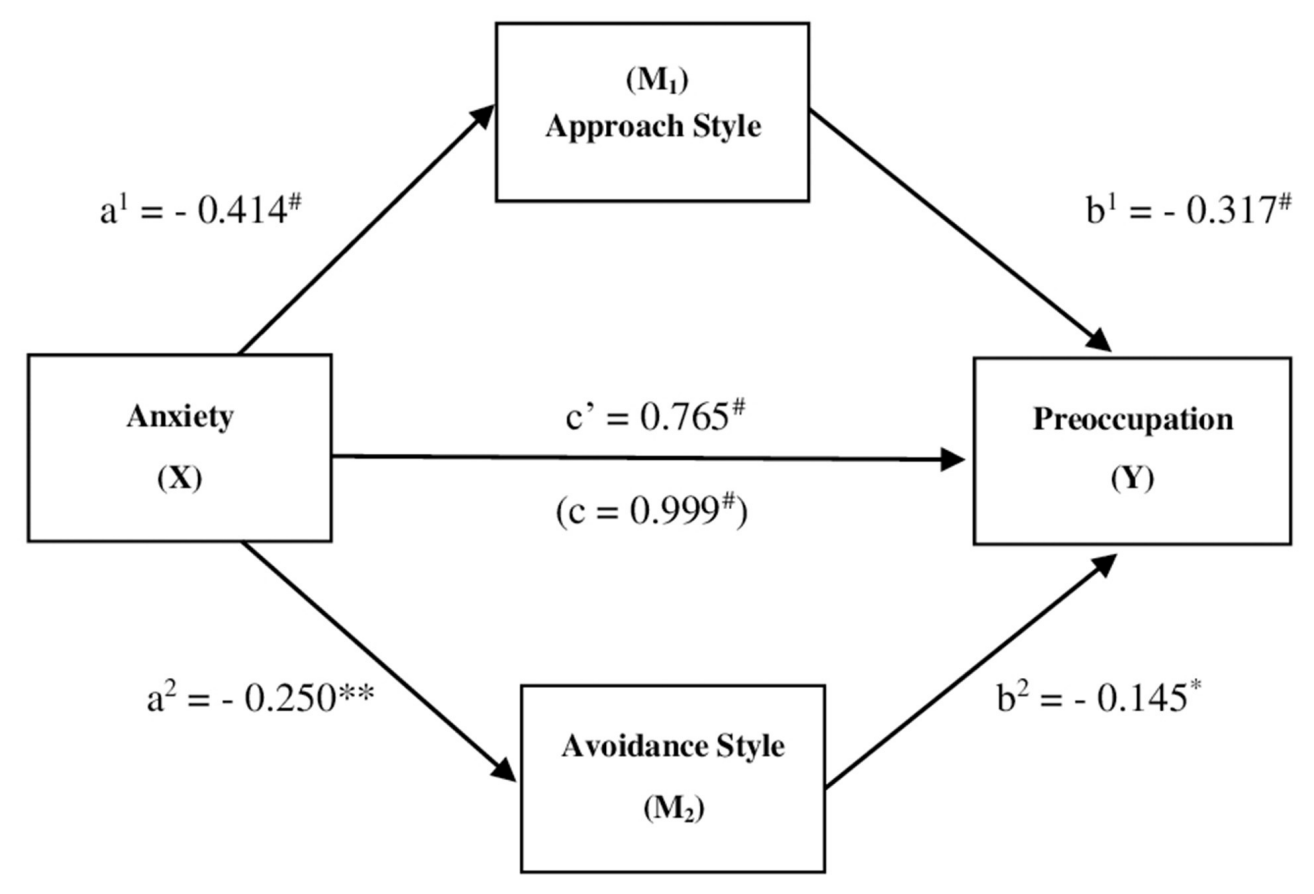
Parallel mediating effect of *Coping Styles* in the relationship between *Anxiety* and *Pre-occupation*. ^#^*p* < 0.001; **p < 0.01; *p < 0.05; all presented effects are standardized; a^1^ is effect of Anxiety on Approach Style; b^1^ is effect of Approach Style on Pre-occupation; a^2^ is effect of Anxiety on Avoidance Style; b^2^ is effect of Avoidance Style on Pre-occupation; c' is direct effect of Anxiety on Pre-occupation, c is total effect of Anxiety on Pre-occupation.

For Athlete's Pre-occupation a parallel mediation of Approach Style and Avoidance Style on the effect of the Anxieties was hypothesized. In this respect, the three variables of anxiety (somatic, social and concentration disorder) have been aggregated into a single variable of Anxiety. Results from the parallel mediation analysis, as shown in Figure 1, indicated that Anxiety was indirectly related to Pre-occupation through its relationships with the Approach Style and the Avoidance Style. First, as can be seen in Figure 1, Anxiety had a negative effect on the Approach Style (*a*^1^ = −0.414, *p* = 0.000), and a higher reported Approach Style was subsequently related to less Pre-occupation (*b*^1^ = −0.317, *p* = 0.000). A 95% bias-corrected confidence interval based on 10,000 bootstrap samples indicated that the indirect effect through Approach Style (*a*^1^*b*^1^ = 0.131), holding the other mediator constant, was entirely above zero (0.060 to 0.212). Secondly, Anxiety had a negative effect on Avoidance Style (*a*^2^ = −0.250, *p* = 0.007), and a higher reported Avoidance Style was subsequently related to less Pre-occupation (*b*^2^ = −0.145, *p* = 0.018). A 95% bias-corrected confidence interval based on 10,000 bootstrap samples indicated that the indirect effect through Avoidance Style (*a*^2^*b*^2^ = 0.036), holding the other mediator constant, was entirely above zero (0.003 to 0.077). Moreover, higher levels of Anxiety corresponded to higher Pre-occupation even after taking into account Anxiety's indirect effect through Approach Style and Avoidance Style (*c'* = 0.765, *p* = 0.000). The total indirect effect was 0.168 [0.090; 0.248], while (C1) Approach Style *minus* Avoidance Style = 0.095 [0.010; 0.191].

## Effects of Anxieties and Coping Styles on Athlete's Decentering Competence

A hierarchical multiple regression was run to determine if the addition of components of Anxiety and Coping Styles improved the prediction of the Athlete's Decentering. Influential predictors have been identified in Somatic Anxiety (β = −0.195; Δ*R*^2^ = 0.193), Social Anxiety (β = −0.216; Δ*R*^2^ = 0.063), Avoidance Style (β = 0.211; Δ*R*^2^ = 0.043), Concentration Disorder (β = −0.190; Δ*R*^2^ = 0.025). The full model to predict Athlete's Decentering was statistically significant, *R*^2^ = 0.324, *F*_(4, 114)_ = 13.201, *p* < 0.0005; adjusted *R*^2^ = 0.300.

## Avoidance Style as Mediator of Anxieties on Decentering

**Figure 2:**
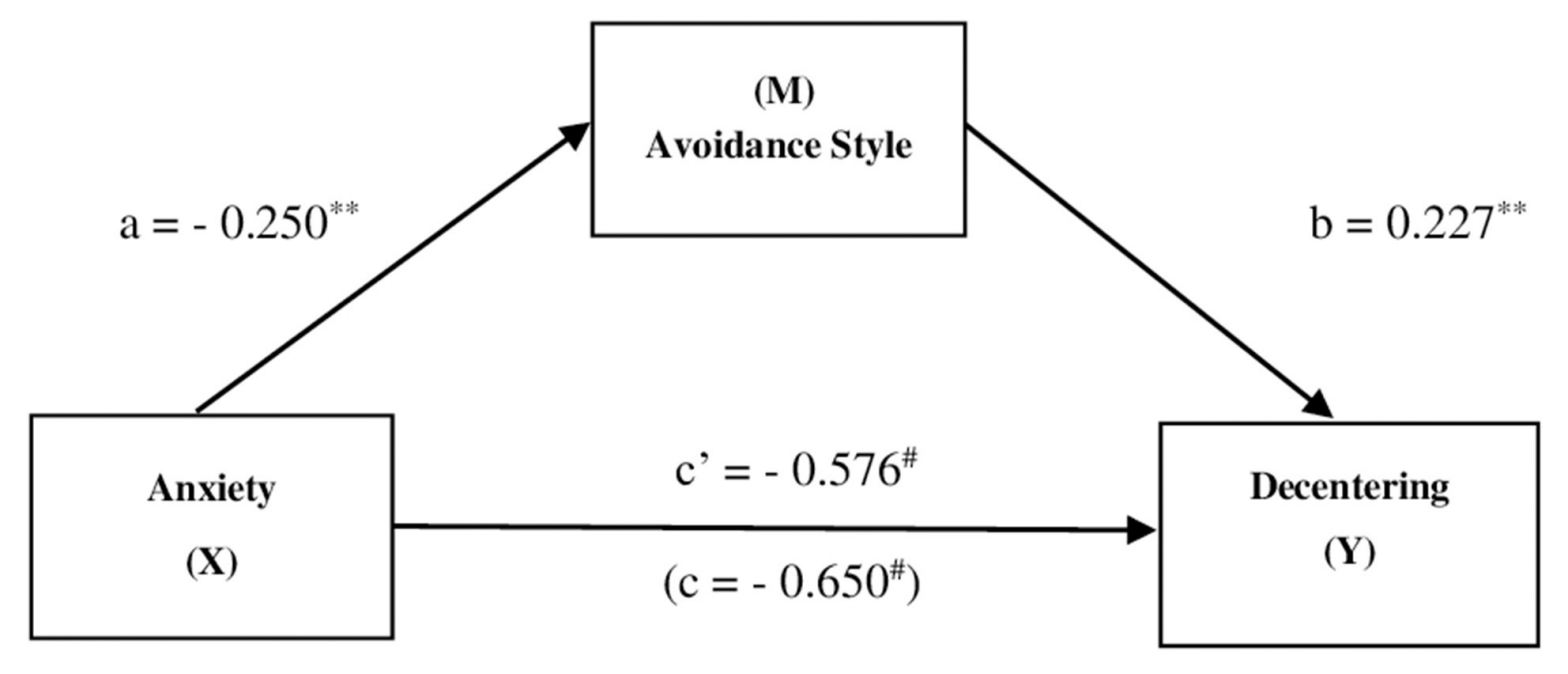
The mediating effect of *Avoidance Style* in the relationship between *Anxiety* and *Decentering*. **p < 0.01; ^#^*p* < 0.001; all presented effects are standardized; a is effect of Anxiety on Avoidance Style; b is effect of Avoidance Style on Decentering; c' is direct effect of Anxiety on Decentering, c is total effect of Anxiety on Decentering.

For Athlete's Decentering a simple mediation of Avoidance Style on the effect of the Anxieties was hypothesized. In this respect, the three variables of anxiety (somatic, social and concentration disorder) have been aggregated into a single variable of Anxiety. Results from the simple mediation analysis, as shown in Figure 2, indicated that Anxiety was indirectly related to Decentering through its relationship with Avoidance Style. First, as can be seen in Figure 2, Anxiety had a negative effect on the Avoidance Style (*a* = −0.250, *p* = 0.007), and higher reported Avoidance Style was subsequent related to more Decentering (*b* = 0.227, *p* = 0.006). A 95% bias-corrected confidence interval based on 10.000 bootstrap samples indicated that the indirect effect (*ab* = −0.060) was entirely below zero (−0.17 to −0.011). Moreover higher levels of Anxiety corresponded to lower Decentering even after taking into account Anxiety's indirect effect through Avoidance Style (*c'* = −0.576, *p* = 0.000).

## Effects of Athlete's Pre-Occupation, Decentering and Coach Support on the Frequency of Choking

A hierarchical multiple regression was run to determine if the addition of components of Pre-occupation, Decentering and Coach Support improved the prediction of the Choking. Influential predictors have been identified in *Pre-occupation* (β = 0.230; Δ*R*^2^ = 0.101), *Coach Support* during Competition (β = −0.231; Δ*R*^2^ = 0.046), *Decentering* (β = −0.250; Δ*R*^2^ = 0.047). The full model of Pre-occupation, Coach Support, Decentering to predict *Frequency of Choking* was statistically significant, *R*^2^ = 0.193, *F*_(3, 114)_ = 8.876, *p* < 0.0005; adjusted *R*^2^ = 0.172.

### A Hypothesis of Structural Model Explaining Choking Frequency in Archers

**Figure 3:**
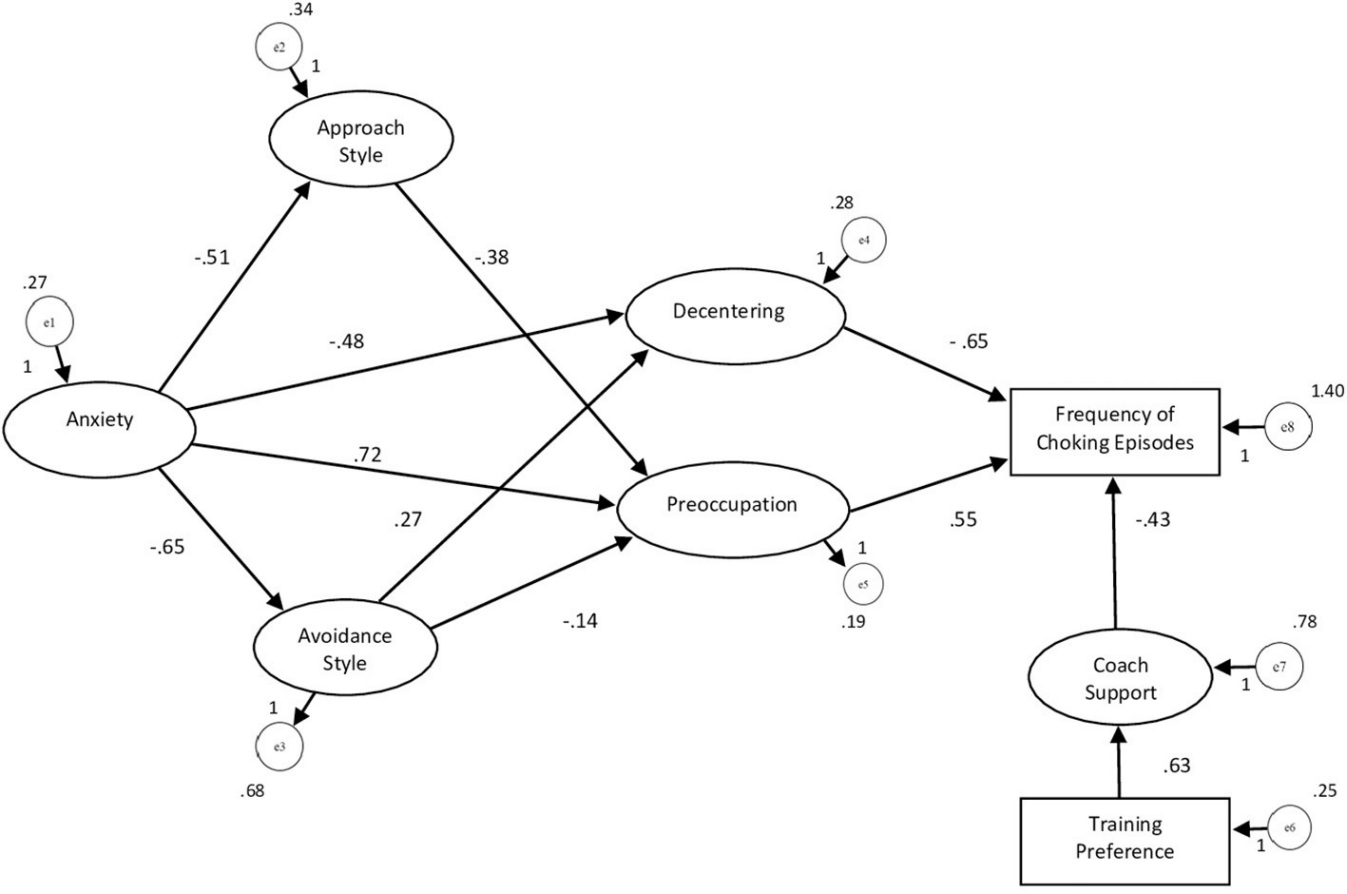
Structural explanatory modal of Archer's Choking Frequency. χ^2^ = 21.974; DF = 17; *p-*value = 0.186; RMSEA = 0.51.

Subsequently a SEM analysis was performed combining in one explanatory model both the mediation analyses with the variables that previously revealed significant association with the frequency of choking. Furthermore, the two variables of perceived support of one's coach in competition and in training have been merged into one single variable (Coach), while Training Preference was recoded with 0 for “alone” and 1 for “in group” (see Figure 3).

The model showed overall good fit measurements: χ^2^ = 21.974; DF = 17; *p* = 0.186; CMIN/DF = 1.293; RMR = 0.052; GFI = 0.964; AGFI = 0.906. Baseline Comparisons NFI = 0.917; IFI = 0.980; CFI = 0.979. Parsimony-Adjusted Measures PNFI = 0.557; PCFI = 0.594; RMSEA = 0.051; PCLOSE = 0.450; RMSEA 90% = 0.000 −0.105.

The model showed that Frequency of Choking was firstly affected by Pre-occupation (standardized estimate of the regression weight of 0.227 for *p* < 0.018). The archer's ability to decentralize instead showed a significant negative effect (standardized estimate of the regression weight of −0.229 for *p* < 0.007), while a further negative effect on the frequency of choking resulted from the support of the own coach perceived (standardized estimate of the regression weight of −0.248 for *p* < 0.010).

Pre-occupation has been found influenced by the effects of the anxiety components (social anxiety, somatic anxiety, concentration disturbance) (standardized estimates of the regression weights 0.515 for *p* < 0.001). Anxiety had negative effects on Coping Styles, respectively standardized estimates of the regression weights −0.414 on *Approach Style* with *p* < 0.001, and −0.379 through *Avoidance Style* with *p* < 0.001. Moreover, Coping Styles had negative effects on *Pre-occupation* (standardized estimates of the regression weights respectively −0.169 with *p* < 0.01 through *Avoidance Style*; −0.334 with *p* < 0.001 through *Approach Style*) and on *Decentering* (standardized estimates of the regression weights, respectively 0.358 with *p* < 0.001 through *Avoidance Style*. Finally, the preference for group training (rather than alone) has shown to influence the *Support Perceived by one's Coach* (standardized estimates of the regression weights 0.335 for *p* < 0.001). In the complete model it is possible to find confirmation to the two mediation analyses previously verified with PROCESS, that is the parallel mediation of the Coping Styles on the effects of Anxiety on Pre-occupation and the simple mediation of the Avoidance Style on the effects of Anxiety on the Decentering ability of the archer. The following tables summarize the *Maximum Likelihood Estimates* (Table 3) and the *Regression Weights Estimates* (Table 4).

**Table 3:** Maximum likelihood estimates.

**Label**		**Label**	**Estimate**	**S.E**.	**C.R**.	***P***
Anxiety	⇒	Approach Style	−0.509	0.105	−4.856	***
Anxiety	⇒	Avoidance Style	−0.650	0.149	−4.370	***
Avoidance Style	⇒	Pre-occupation	−0.137	0.050	−2.738	0.006
Approach Style	⇒	Pre-occupation	−0.378	0.071	−5.333	***
Anxiety	⇒	Pre-occupation	0.718	0.092	7.807	***
Avoidance Style	⇒	Decentering	0.268	0.061	4.433	***
Anxiety	⇒	Decentering	−0.475	0.104	−4.571	***
Training Preference	⇒	Coach Support	0.632	0.167	3.797	***
Pre-occupation	⇒	Choking	0.550	0.233	2.359	0.018
Coach Support	⇒	Choking	−0.650	0.252	−2.576	0.010
Decentering	⇒	Choking	−0.427	0.158	−2.707	0.007

*** *p ≤ 0.001*.

**Table 4:** Standardized regression weight estimates.

Anxiety	⇒	Approach Style	−0.414
Anxiety	⇒	Avoidance Style	−0.379
Avoidance Style	⇒	Pre-occupation	−0.169
Approach Style	⇒	Pre-occupation	−0.334
Anxiety	⇒	Pre-occupation	0.515
Avoidance Style	⇒	Decentering	0.358
Anxiety	⇒	Decentering	−0.369
Training Preference	⇒	Coach Support	0.335
Pre-occupation	⇒	Choking	0.227
Coach Support	⇒	Choking	−0.248
Decentering	⇒	Choking	−0.229

## Discussion

This study aimed to test the fit of an explanatory model of the frequency of the phenomenon of choking under pressure in archers. Through the graphical representation of the model we could observe that cognitive component of archer's pre-occupation was the main predictive element for choking. Two other components of the model have been shown to have limiting effects on choking: the ability to decentralize and the coach's perception of support.

Psychologically, pre-occupation is part of persevering cognition, which implies thinking continuously about negative events in the past or future (see Hirsch and Mathews, 2012). Overly concerned athletes overestimate future dangers in their evaluations and tend to exaggerate the situation in a vicious circle that causes stress (Brosschot et al., 2005). Chronically concerned people are likely to lack confidence in their ability to solve problems, perceive problems as threats, feel easily frustrated when facing a problem and are pessimistic about the outcome of efforts to try to solve it. When concern becomes excessive and beyond a person's control, the latter spends most of his or her time thinking and brooding about what is causing concern; so much so that he or she feels crushed and trapped by these thoughts, making the brooding pathological (Bredemeier and Berenbaum, 2008; Zozulya et al., 2008).

According to studies carried out by Borkovec et al. (1983, 2004), the fundamental characteristics of brooding are the predominance of negative verbal thinking, to the detriment of imaginative thinking, cognitive avoidance and inhibition of emotional elaboration. This behavior can become maladaptive as a repeated condition of emotional inhibition over time can lead to a stability of unpleasant emotions. Remembrance, therefore, is a continuous mental repetition of the fear of uncontrollable harm without the representation of concrete scenarios of implementation. Therefore, brooding is ineffective and unproductive (Ruscio et al., 2001; Ruscio and Borkovec, 2004).

Borkovec and Inz (1990) consider that the people who implement brooding attribute to this mode of thinking advantages and purposes that reinforce the brooding itself. Among these are: reducing anxiety (but this mode of thinking does nothing but maintain anxiety); resolving situations (in reality it is not so because the person remains firm and rigid, does not elaborate concrete and effective alternatives); “emotional shield,” that is, the person thinks that the brooding keeps him in a state of alarm and therefore keeps him ready to face the feared situation; ascopic brooding, the subjects are not able to explain the reasons why they brood, so the brooding seems to have no precise purpose and for this reason it is seen as something uncontrollable from which it is difficult to escape. Brooding is a construct of rumination and it can be associated to anxiety, depressive symptoms and psychological distress (Michael et al., 2007; Egan et al., 2013; Tahtinen et al., 2019).

Chronic brooding also has influences on physical and mental health, as it causes cardiovascular problems, muscle tension, insomnia, interpersonal problems. Seriously anxious people have difficulty controlling their anxiety and generally show symptoms such as restlessness, fatigue, difficulty concentrating, irritability, muscle tension and sleep disorders (Watkins and Roberts, 2020).

Brandt et al. (2017, 2019) showed that for elite athletes during the stressful competition period, perceived sleep quality and mood states have significant additive and predictive effects on performance. Both poor sleep quality and depressive states have been shown to negatively affect athletes' competition outcomes.

The model developed in the paper indicated that the archer's worries are directly triggered by other anxiety components, such as somatic and social anxiety and concentration disorder, but at the same time they are mediated by the athlete's coping style. In particular, the Approach Style appears to have the greatest negative effect on the athlete's brooding activity.

Coping, meant as the set of mental and behavioral strategies that are implemented to cope with a certain situation, has traditionally been considered as a rather stable personality characteristic (Carver et al., 1989; Vollrath, 2001). Subsequently, coping modalities have been analyzed as flexible and changeable reactions to regulatory challenges or stressful everyday life events. These processes are considered cyclical and cumulative, therefore the different components shape each other over time and the results obtained from time to time influence the repertoire and coping resources available to the individual to negotiate subsequent interactions and stressful situations (Maas and Spinath, 2012).

Moos and Schaefer (1993) proposed a typology of coping styles that derives from the intersection of tendency to approach vs. avoidance and tendency to engage in a cognitive effort vs. behavioral effort. From the combination of these possibilities four basic modes of coping emerge coping: (a) of cognitive approach, based on strategies of rational analysis of the situation or of (b) a behavioral approach, based on the tendency to implement actions aimed at changing the stressful situation; (c) avoidance-cognitive, which consists of denying or minimizing the severity of the stressful event or its effects; and (d) of behavioral-avoidance, based on the implementation of other forms of satisfaction that may replace the losses resulting from the crisis situation.

Avoidance coping has traditionally been considered a maladaptive strategy (Zeidner and Saklofske, 1996). However, some research has more recently highlighted its potential functionality. It has been in fact, noted that when faced with uncontrollable events and their consequences in the short term, this coping mode is a very effective way of managing the situation (Many et al., 2012).

A study on netball players also showed that an avoidance coping strategy was adopted during successful performances (Mesagno and Marchant, 2013) but this result is in contrast with Hill et al. (2010) that, in a study of elite golfer, showed that regular choking under pressure was associated with the strategy of the attempt to avoid stressful situations.

The line of studies on coping approach has highlighted the functionality of a new type of coping, called proactive (Aspinwall and Taylor, 1997). This mode embodies a way of dealing with stress altogether particular, aimed more at its prevention than at the response following its deployment. Coping proactive therefore represents a set of strategies based on the acquisition of resources personal, which facilitate the ability to prevent critical events, and on the promotion of the individual development, e.g., through planning strategies, choice and active guidance of the life goals that have proven to be very effective in avoiding situations that are excessively stressful (Aspinwall, 2005; Ruiselova and Prokopcakova, 2010).

In our model to understanding archer's choking, it was found that approach coping can limit the brooding tendency. The approach coping involves confronting the source of stress and trying to deliberately reduce it through strategies that include direct action, increased effort and planning (see Roth and Cohen, 1986). The appraisal-oriented approach involves re-evaluating a situation to reduce its importance and refers to strategies such as restructuring the situation (Cox and Ferguson, 1991). Problem focused coping, in this sense, includes several subtypes of coping, such as information seeking, planning and goal setting, and assertive confrontation. Examples of emotionally focused management include elements such as seeking emotional support, relaxation or meditation and desire. Avoidance coping, on the other hand, includes both behavioral efforts (e.g., eliminating oneself from the situation) and psychological efforts (e.g., cognitive distancing) to disengage oneself from a stressful situation (see Krohne, 1993).

Avoidance has shown in the model that it can also partly help to limit the effects of anxiety on concern, but its greater effect is that of positive mediation than the athlete's ability to decentralize. Decentering and defusion corresponds to the ability to observe with detachment one's thoughts, emotions and feelings as passenger events, which provide information but to which it is not strictly necessary to react or try to control them (Bernstein et al., 2015). These objectives are aimed at establishing specific mechanisms of action, determining for an optimal performance and at the same time for the well-being of the athlete: to increase emotional regulation (not emotional control) through acceptance and decentralization; to increase executive functioning through greater awareness and attention; to encourage a committed behavior, directed to the task, at the service of personal goals and values (McCracken et al., 2014).

Decentering, the ability to observe one's thoughts and feelings from a detached view, has gained increased attention in recent years even to avoid choking (Jones et al., 2009; Zhang et al., 2016). Decentering is a key construct that is related to individuals' adaptive and maladaptive psychological constructs (Bernstein et al., 2015). Experimental evidence has supported the protective role of decentering in that, even with high levels of rumination, individuals high in decentering produced better task performance when exposed to interpersonal criticism (see Kaiser et al., 2015, cited in Zhang et al., 2016). In sport, one important aim of mindfulness training is to cultivate athletes' ability to decenter from previously formed automatic connections among thoughts, feelings, and behavioral choices (Gardner and Moore, 2004). In mindfulness training, athletes are encouraged to view their thoughts as simply passing events that may or may not accurately reflect the realities around them, and the decentering ability is produced accordingly (Gardner and Moore, 2007).

In our study, the gender was also associated indirectly to the occurrence of choking under pressure. Female athletes registered higher values of pre-occupation and lower levels of decentralization skills, both variables associated with choking episodes. This result is consistent with the findings of a research on university athletes (Adegbesan, 2007) but not with researches on professional tennis players where, even if women showed a drop in performance in the more crucial stages of the match, it was in any event about 50% smaller than that of men (Cohen-Zada et al., 2017). Gneezy et al. (2003) observed that a gender difference in choking was reported when women competed against men but not when they competed in single-sex environments.

In the model of the study with archers it was found that decentralization contributes significantly, together with the perceived support of one's own coach, to lower the frequency of coaching episodes. Probably the effect attributable to the coach is due to the influence on the athlete's perception of self-efficacy. In a qualitative examination of choking under pressure in team sport, Hill and Shaw (2013) found that the level of social support that the athletes received from their coach could moderate their tendency to choke under pressure. When the coach promoted an educational approach and offered emotional and informational support, the participants reported less frequent episodes of choking. According to Tamminen and Holt (2012), social support facilitates the athlete to cope more effectively with pressure and better manage the stress. In fact, in our model with archers it emerged that those who preferred to train in groups were also those who declared a higher level of perceived support. In this case the positive contribution of the activation of the sense of collective self-efficacy probably also comes into play (Diotaiuti et al., 2017).

The results of the study partially confirm the findings of the previous literature concerning the importance of the anxiety and attention constructs in the choking under pressure process. Among the innovative aspect of this model there are the identification of the mediations that coping styles exert on the effects of anxiety on the two essential predictive components of choking, i.e., cognitive brooding (positive predictor) and the ability to decentralize (negative predictor). The frequency of the episodes of choking has found a significant further negative predictor in the coach technical support perceived by the archer. The importance of the coach assistance during practices and competitions in limiting the choking frequency, as far as we know, has never been included in previous research on archery.

The future research in the archery could better investigate the protective role of the coach and teammates on choking and also the effects on the variable of self-efficacy of the athlete, which in our research had not been considered in the design of the protocol.

Further studies should also deepen the evaluation of the athlete's ability to decentralize and the measurement of the effectiveness of specific training aimed at enhancing this ability in archers.

## Practical Implications

Based on previous research choking under pressure studies in sport, several intervention to prevent the phenomenon of choking under pressure in sport have been developed and tested such as self-focus and distraction based interventions, acclimation interventions, mindfulness interventions, pre-performance routines, thought stopping, imagery and eye training (Singer, 1988; Hussey, 2015; Gröpel and Mesagno, 2017; Mesagno and Beckmann, 2017). Specific research on anxiety in the archers seems to prefer relaxation intervention to pre performance routine interventions and underlie the importance of identifying and evaluating needs and assessment of the athlete (Cotterill et al., 2016). The results of our study indicate three essential areas of attention and intervention: first of all, the careful assessment of the athlete's coping style in situations of stress and pressure, given the important role of mediation on the rimuginative or self-regulatory (decentralizing) response to the athlete's anxiety; differentiated interventions on these two levels aimed at blocking expressions of remorse and enhancing the ability to decentralize; greater involvement and encouragement of the coach to provide systematic support both in training and competition. His role in this case could also be that of promoter in the athlete of what Folkman and Moskovitz (2000) called “coping focused on meaning,” stimulating in the athlete under pressure the ability to find benefits in the negative event (for example a personal maturation or a strengthening of interpersonal relationships), the ability to keep these benefits in mind, the ability to change one's goals when stressful circumstances threaten or compromise one's current goals, the ability to re-weight one's priorities, changing ones vision of what is more or less important, and the ability to assess normal or ordinary life events as positive (Folkman, 2008). In such a case, the presence and active support of the coach and/or the team group would limit the use of maladaptive coping strategies that risk being a source of stress and unpleasant feelings, leading the athlete to further attempts at avoidance, followed by the need to cope with new stress factors (Littleton et al., 2007).

## Strengths and Limitations

Considering previous research on archery, the sample size of our study is a strength, besides that most of them competing in the first category – the most competitive, involving elite athletes of the Italian Archery Federation. Furthermore, the importance of the coach assistance during training and competitions in limiting the choking frequency, as far as we know, has never been included in previous research. A potential limitation of the current study was that due to the cross-sectional design, so caution is need regarding the conclusions about causality of performance, since that we did not verified the individual results on the competitions.

## Conclusion

The results of this study highlighted an explanatory model of choking in the archer in which the ruminative component of concern was found to be the factor directly influencing the frequency of choking episodes in performance. Two significant mediations through personal coping style were also identified on the effects of anxiety on brooding thinking and on athlete's ability to decentralize. The latter personal self-regulative component has been shown to be able to strongly limit the frequency of choking under pressure. Finally, among the environmental components, a further facilitating influence for the athlete was found in the perception of being supported by the own coach. The results therefore suggested that the athlete in choking should not face alone the difficulty he is going through, but should preferably be oriented to follow a program focused in the cognitive remodeling of disturbing thoughts, in the strengthening of the capacity of decentralization, in the enhancement of the relationship of support and trust with the coach.

## Data Availability Statement

The raw data supporting the conclusions of this article will be made available by the authors, without undue reservation.

## Ethics Statement

Ethical review and approval was not required for the study on human participants in accordance with the local legislation and institutional requirements. Written informed consent to participate in this study was provided by the participants, and where necessary, the participants' legal guardian/next of kin.

## Author Contributions

PD, SC, and SM designed the study, analyzed the data, and discussed the results. PD, SC, LF, FD, and AA drafted the manuscript. LF, FD, and AA revised the manuscript. All authors approved the final manuscript. All authors have agreed to be accountable for all aspects of the manuscript in ensuring that questions related to the accuracy or integrity of any part of it are appropriately investigated and resolved.

## Conflict of Interest

The authors declare that the research was conducted in the absence of any commercial or financial relationships that could be construed as a potential conflict of interest.
